# Transport Mechanisms and Their Pathology-Induced Regulation Govern Tyrosine Kinase Inhibitor Delivery in Rheumatoid Arthritis

**DOI:** 10.1371/journal.pone.0052247

**Published:** 2012-12-20

**Authors:** Christian Schmidt-Lauber, Saliha Harrach, Thomas Pap, Meike Fischer, Marion Victor, Marianne Heitzmann, Uwe Hansen, Manfred Fobker, Stefan-Martin Brand, Aleksandra Sindic, Hermann Pavenstädt, Bayram Edemir, Eberhard Schlatter, Jessica Bertrand, Giuliano Ciarimboli

**Affiliations:** 1 Experimental Nephrology, Department of Internal Medicine D, University Hospital Münster, Münster, Germany; 2 Institute of Experimental Musculoskeletal Medicine, University Hospital Münster, Münster, Germany; 3 Institute for Physiological Chemistry and Pathobiochemistry, University Hospital Münster, Münster, Germany; 4 Center of Laboratory Medicine, University Hospital Münster, Münster, Germany; 5 Leibniz-Institute for Arteriosclerosis Research, University Hospital Münster, Münster, Germany; 6 Molecular Genetics of Cardiovascular Disease, Institute of Sports Medicine, University Hospital Münster, Münster, Germany; 7 Department of Physiology, Croatian Institute for Brain Research, University of Zagreb, Zagreb, Croatia; Royal College of Surgeons, Ireland

## Abstract

**Background:**

Tyrosine kinase inhibitors (TKIs) are effective in treating malignant disorders and were lately suggested to have an impact on non-malignant diseases. However, in some inflammatory conditions like rheumatoid arthritis (RA) the *in vivo* effect seemed to be moderate. As most TKIs are taken up actively into cells by cell membrane transporters, this study aimed to evaluate the role of such transporters for the accumulation of the TKI Imatinib mesylates in RA synovial fibroblasts as well as their regulation under inflammatory conditions.

**Methodology/Principal Findings:**

The transport and accumulation of Imatinib was investigated in transporter-transfected HEK293 cells and human RA synovial fibroblasts (hRASF). Transporter expression was quantified by qRT-PCR. In transfection experiments, hMATE1 showed the highest apparent affinity for Imatinib among all known Imatinib transporters. Experiments quantifying the Imatinib uptake in the presence of specific transporter inhibitors and after siRNA knockdown of hMATE1 indeed identified hMATE1 to mediate Imatinib transport in hRASF. The anti-proliferative effect of Imatinib on PDGF stimulated hRASF was quantified by cell counting and directly correlated with the uptake activity of hMATE1. Expression of hMATE1 was investigated by Western blot and immuno-fluorescence. Imatinib transport under disease-relevant conditions, such as an altered pH and following stimulation with different cytokines, was also investigated by HPLC. The uptake was significantly reduced by an acidic extracellular pH as well as by the cytokines TNFα, IL-1β and IL-6, which all decreased the expression of hMATE1-mRNA and protein.

**Conclusion/Significance:**

The regulation of Imatinib uptake via hMATE1 in hRASF and resulting effects on their proliferation may explain moderate *in vivo* effects on RA. Moreover, our results suggest that investigating transporter mediated drug processing under normal and pathological conditions is important for developing intracellular acting drugs used in inflammatory diseases.

## Introduction

Tyrosine kinases play a critical role in signaling driven by growth factors and oncoproteins thus regulating cellular key functions like proliferation and cell death. Uncontrolled tyrosine kinase signaling is known to be associated with various malignancies. Tyrosine kinase inhibitors (TKIs) have a tremendous effect in chemotherapeutic treatment and revolutionized the treatment of chronic myelogenous leukemia (CML). However, modified tyrosine kinase signaling is also associated with non-malignant disorders such as fibrotic and inflammatory diseases [1]. Several *in vitro* studies showed promising effects of TKIs on systemic sclerosis (SSc), rheumatoid arthritis (RA) and other fibrotic diseases by inhibition of TGFβ and PDGF signaling [2], [3]. In addition to possible effects on autoimmunity [4], the TKI Imatinib mesylates (Gleevec®) was shown to inhibit PDGF mediated proliferation of synovial fibroblasts (SF) and reduce fibrogenesis and activation of fibroblast-like synoviocytes in RA [5]–[8]. However, in contrast to their use in malignant disorders, TKIs could not always fulfill promising *in vitro* effects on inflammatory diseases *in vivo.* This becomes apparent by the fact that nearly no clinical studies are available for TKI treatment of SSc and RA. As the targeted kinases are similar in benign and malignant disorders, this observation is unlikely due to a different way of interaction with their targets. Most TKIs are actively transported into targeted cells, as they are poorly lipophilic and cannot passively pass the cell membrane. This uptake process is well known to regulate the efficacy of these drugs and its importance has been shown for the best-analyzed TKI Imatinib. Its uptake in leukocytes, the target cells in CML treatment, directly regulates the efficacy of Imatinib [9]. The human organic cation transporter 1 (hOCT1) has been suggested to mediate leukocyte uptake [10]. Lately, further transporters have been proposed to interact with Imatinib; among them the human multidrug and toxin extrusion transporter 1 (hMATE1) [11].

A main target of Imatinib and other TKIs in RA are synovial fibroblasts (SF) as they potently mediate synovial hyperplasia leading to joint destruction [12]. To date, the mechanisms by which fibroblasts accumulate TKIs are unknown. This study aims to evaluate the importance of this transport process for the delivery of TKIs in RA and its pathology induced regulation exemplary for Imatinib.

## Results

### hMATE1 Transports Imatinib with Higher Affinity than other OCT

**Figure 1:**
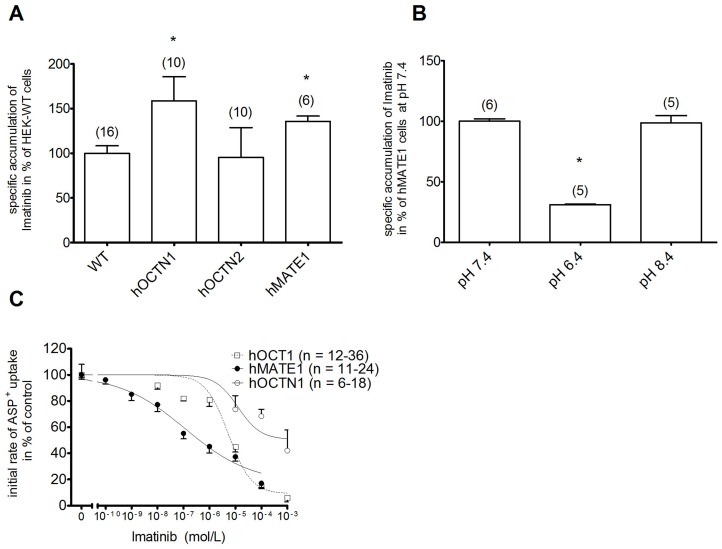
Properties of Imatinib transport by hOCTN1, hOCTN2 and hMATE1. A) Specific Imatinib uptake (10 µM) in transfected HEK293 cells given as difference of accumulation at 4°C and at 37°C measured by HPLC. B) Effect of extracellular pH on the specific Imatinib uptake (10 µM) in hMATE1 transfected HEK293 cells. C) Apparent affinities of Imatinib on hOCT1 (IC_50_ = 5 µM), hOCTN1 (IC_50_ = 31 µM) and hMATE1 (IC_50_ = 118 nM) measured by concentration dependent inhibition of ASP^+^ uptake. Results show number of observation in brackets. Values are mean ± SEM. * indicates statistically significant effects (P<0.05).

To find out whether other transporters than hOCT1 are responsible for Imatinib delivery in RA, we investigated its transport by hOCTN1, hOCTN2 and hMATE1. Therefore, the uptake of transfected HEK293 cells was compared to WT-HEK293 cells having an absolute transport rate of 0.19±0.02 fmol Imatinib/cell (n = 16) which is set to 100%. Both, hOCTN1 and hMATE1 are able to translocate Imatinib as HEK293 cells that were transfected with these transporters showed a significantly higher accumulation than WT-HEK293 cells (+59±27%, n = 10 for hOCTN1 and +36±6%, n = 6 for hMATE1, Fig. 1A). In contrast, hOCTN2 transfected HEK293 cells did not significantly differ in the Imatinib uptake from WT-HEK293 cells (Fig. 1A), indicating no significant transport by hOCTN2. According to its properties as H^+^/organic cation antiporter, the transport by hMATE1 was pH dependent since it decreased by 69±1% (n = 5) when the extracellular pH was set to 6.4 (Fig. 1B). Next, we compared the apparent affinities of hOCT1, hMATE1 and hOCTN1 for Imatinib by inhibiting the uptake of their model substrate ASP^+^ (Fig. 1C). Calculated IC_50_ values revealed a remarkably higher apparent affinity of Imatinib to hMATE1 (IC_50_ = 21 nM) than to hOCT1 (IC_50_ = 5 µM) and hOCTN1 (IC_50_ = 31 µM). Experiments for hMATE1 were also performed at an extracellular acidic pH showing a severely decreased apparent affinity (Fig. S3).

### hMATE1 Mainly Mediates Imatinib Uptake in hRASF

**Figure 2:**
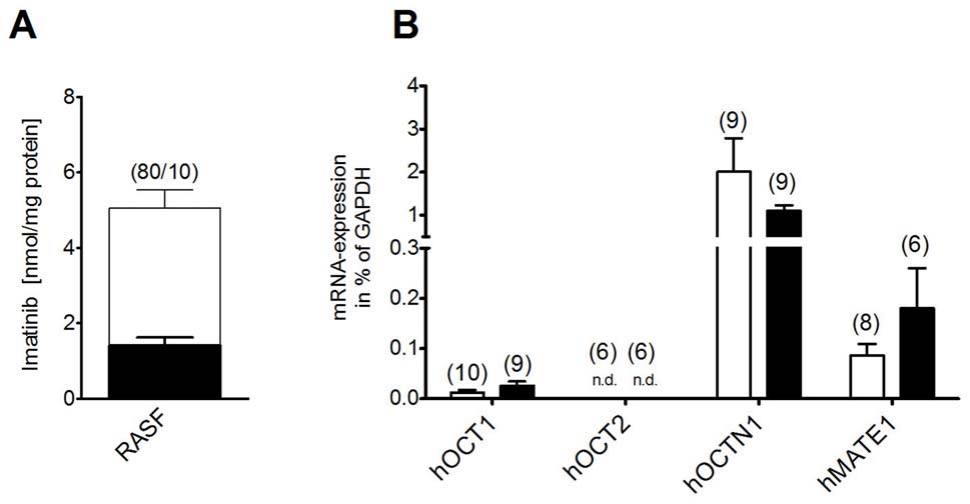
hRASF actively accumulate Imatinib and express several OCTs. A) Temperature dependent uptake of Imatinib (10 µM) in hRASF at 4°C (black column) and 37°C (white column) measured by HPLC. B) Expression of investigated transporters in hRASF (white columns) and hOASF (black columns) determined by qRT-PCR. Values are mean ± SEM. n.d. = not detected.

First we investigated whether hRASF are capable to actively accumulate Imatinib by quantifying their uptake via HPLC. The significantly higher uptake at 37°C (3.6±0.5 nmol/mg protein, n = 10) than under inhibition of metabolic processes at 4°C (1.4±0.2 nmol/mg protein n = 10) indicates a transporter-mediated process (Fig. 2A). Thus, the expression of potential Imatinib transporters (hOCT1, hOCT2, hOCTN1 and hMATE1) in hRASF was quantified by qRT-PCR and compared to hOASF (Fig. 2B). hOCT1 is expressed at low levels in hRASF (0.012±0.005% of GAPDH, n = 10) and hOCT2 mRNA is not detectable in any sample. In contrast, hOCTN1 (2.0±0.8%, n = 9) is expressed at a higher level. hMATE1-mRNA was found to be expressed relevantly in SF with a lower expression in hRASF (0.09±0.02%, n = 8) than hOASF (0.18±0.08%, n = 6), although the difference in the expression did not reach a significant level.

**Figure 3:**
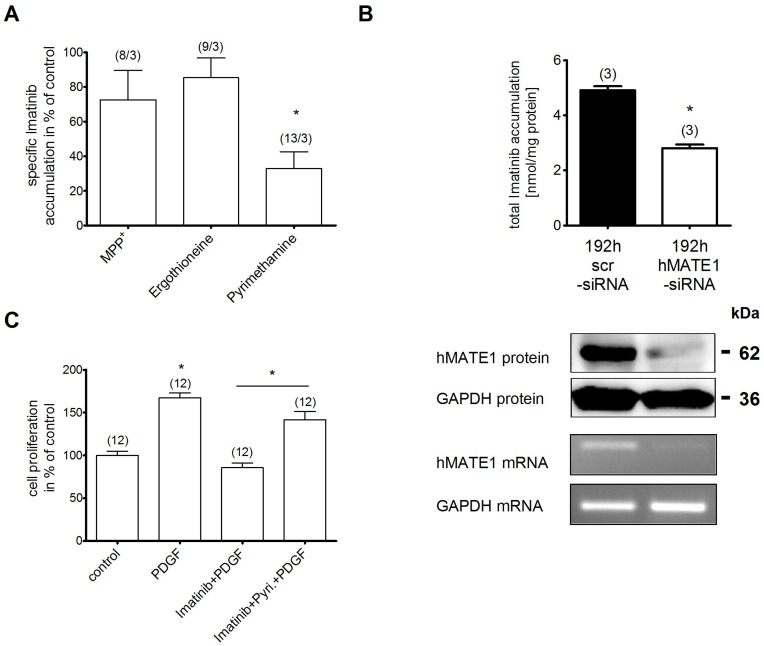
hMATE1 mediates the Imatinib uptake in hRASF and governs anti-proliferating effects. A) Specific uptake of Imatinib (10 µM) in hRASF given as difference of accumulation at 4°C and 37°C with inhibition of hMATE1 (by 200 nM pyrimethamine), hOCT1 (by 20 µM MPP^+^) or hOCTN1 (by 40 µM ergothioneine). Data are given as percentage of uptake without inhibition. B) Specific Imatinib uptake, hMATE1 Western Blot and PCR in hRASF after transfection with hMATE1- or scrambled (scr)-siRNA for 72 and 192 hours. Number of transfections is given in brackets. C) Proliferation on hRASF quantified by cell counting after stimulation with PDGF in the presence and absence of Imatinib and the hMATE1 inhibitor pyrimethamine with number of transfections given in brackets. All values are mean ± SEM. * indicates statistically significant effects (P<0.05).

To test the direct influence of the individual transporters on Imatinib uptake we measured its cellular accumulation in hRASF after co-incubation with specific transporter inhibitors. Inhibition of hOCT1 by MPP^+^ and hOCTN1 by ergothioneine did not change Imatinib uptake (Fig. 3A). In contrast, inhibition of hMATE1 by 200 nM pyrimethamine reduced the Imatinib accumulation in hRASF by 67±10% (n = 3) (Fig. 3A), suggesting that the uptake in hRASF is mainly mediated by hMATE1. These results were confirmed by quantifying the uptake after a knockdown of hMATE1 expression by a specific siRNA (Fig. 3B). Transfection of hRASF with hMATE1 siRNA for 192 h was needed to significantly reduce hMATE1 protein as well as mRNA expression. The down-regulation of hMATE1 resulted in a significant decrease of Imatinib uptake compared to transfection experiments with a scrambled siRNA (2.8±0.1 vs 4.9±0.1 nmol/mg protein, both n = 3) (Fig. 3B). These findings underline the unique importance of hMATE1 for the Imatinib uptake in hRASF.

To further address the relevance of this uptake for therapeutic effects, we investigated its impact on PDGF mediated proliferation in hRASF. As expected, incubation of naive hRASF with PDGF significantly increased cell proliferation by 67±6% (n = 12). Imatinib (2.5 µM) neutralized this effect to baseline proliferation levels (Fig. 3C). This effect of Imatinib was mostly abolished by co-incubation of naive hRASF with pyrimethamine (200 nM), which restored PDGF-induced cell proliferation (+42±9%, n = 12, Fig. 3C). Pyrimethamine alone had no effect on hRASF proliferation (data not shown).

### Inflammatory Conditions Influence Imatinib Uptake and hMATE1 Expression in hRASF

**Figure 4:**
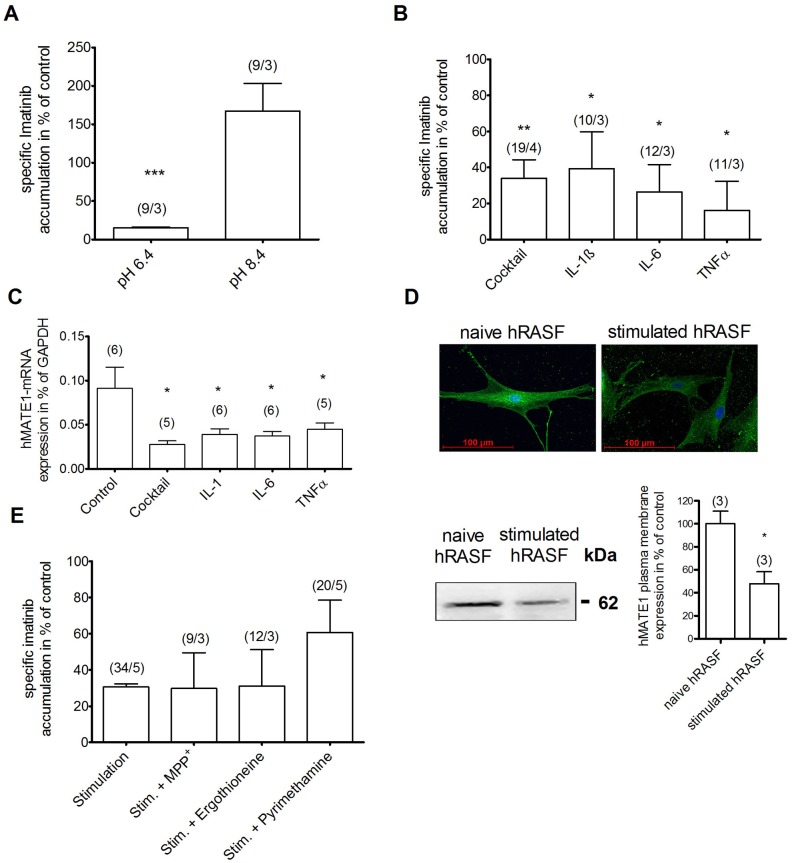
Inflammatory conditions reduce Imatinib uptake and hMATE1 expression in hRASF. Influence of (A) extracellular pH and (B/E) pro-inflammatory cytokines (each at 10 ng/ml) on specific Imatinib uptake (10 µM) given as difference of HPLC detected accumulation at 4°C and 37°C and on (C/D) hMATE1 expression. A) Imatinib uptake in dependence of extracellular pH shown as percentage of uptake at pH 7.4. B, C, and D) Effect of 18 hours incubation with a TNFα, IL-1β and IL-6 (+sIL-6R) cocktail and with single cytokines (each at 10 ng/ml) on Imatinib uptake in hRASF (B), hMATE1-mRNA expression (C), hMATE1-protein expression by immunofluorescence staining (upper part of D) and by Western Blot analysis of biotynilated plasma membrane fractions hMATE1 (lower part of D showing an example of a typical Western blot together with the quantitative analysis of three independent experiments). E) Uptake in hRASF after incubation with cytokine cocktail and inhibition of hMATE1 (by pyrimethamine at 200 nM), hOCT1 (by MPP^+^ at 20 µM) or hOCTN1 (by ergothioneine at 40 µM), measured by HPLC. All values are mean ± SEM. * indicates statistically significant effects (P<0.05).

Since in RA afflicted joints the pH of the synovial fluid becomes acidic [13], its influence on Imatinib uptake was analyzed. Indeed, the uptake in hRASF was pH dependent and decreased at an extracellular acidic pH (Fig. 4A). At pH 6.4 the specific Imatinib uptake was reduced by 85±1% (n = 3), confirming an involvement of a pH dependent transporter such as hMATE1 (Fig. 4A).

As inflammatory cytokines (among them TNFα, IL-1β and IL-6) play a major role in RA pathogenesis [12], their influence on the Imatinib uptake was also examined. Whereas co-stimulation with TNFα, IL-1β and IL-6 for 30 minutes and 12 hours had no effect (Fig. S2), stimulation for 18 hours reduced the specific Imatinib uptake in hRASF by 66±10% (Fig. 4B). To investigate whether only the combination of cytokines or one cytokine alone was responsible for the effect, we stimulated hRASF with single cytokines for 18 hours. Experiments revealed that IL-1β as well as IL-6 (+IL-6R) or TNFα similarly reduced Imatinib uptake by 61±20%, 74±15% and 84±16%, respectively (Fig. 4B). To reveal the mechanism underlying this effect we quantified hMATE1-mRNA after 18 hours stimulation with the cytokine cocktail and single cytokines since hMATE1 was shown to be mainly responsible for this process. hMATE1-mRNA was down-regulated by 70±4% after incubation with the cytokine cocktail and again a similar down-regulation of hMATE1-mRNA was found when incubated with IL-1β (−57±7%), IL-6 (+IL-6R) (−60±6%) or TNFα (−51±8%) alone (Fig. 4C). hMATE1 decrease was also seen on protein level as immunofluorescence staining revealed a remarkably lower expression in hRASF after stimulation with the cytokine cocktail than in naïve hRASF (Fig. 4D). This down-regulation was confirmed by Western blot analysis of plasma membrane fractions isolated during biotinylation experiments. Stimulation of hRASF with the cytokine cocktail revealed a significantly decreased hMATE1 surface expression (−52±10% of naive hRASF, n = 3, Fig. 4D). To confirm that the cytokine induced decrease in Imatinib uptake is due to a reduction of hMATE1 expression, single transporters were blocked with their specific inhibitors after 18 hours incubation with the cytokine cocktail. Again, neither inhibition of hOCT1 by MPP^+^ nor hOCTN1 by ergothioneine changed the uptake (Fig. 4E). Moreover, in contrast to unstimulated hRASF, inhibition of hMATE1 by pyrimethamine had no influence on the intracellular Imatinib amount after stimulation with the cytokine cocktail (Fig. 4E). These data confirm that the down-regulation of Imatinib uptake by cytokines is due to a decrease of hMATE1 expression.

## Discussion

Imatinib and other TKIs have been proposed as therapeutic options in non-malignant disorders including RA [1]. *In vitro* TKIs were shown to inhibit PDGF induced proliferation of hRASF and reduce fibrogenesis and activation of fibroblast-like synoviocytes in RA by interfering with TGFβ and PDGF signaling [5]–[8]. Recently, effects of Imatinib on T cells have also been proposed to play a role for its impact on RA [4]. However, the lack of major clinical studies suggests that the *in vivo* and clinical effects of TKIs on inflammatory diseases like RA are rather moderate. This study gives an explanation for this observation by evaluating the transport of TKI into their targeted cells, in this case for Imatinib. An important target in RA are synovial fibroblasts as they play an important role in the pathogenesis by contributing to joint destruction and producing cytokines [12]. Several transporters which are capable to translocate Imatinib, among them hOCTN1 and hMATE1, which this study newly identifies to transport Imatinib (Fig. 1A), are expressed in hRASF. hMATE1 was shown to have an about 200 fold higher apparent affinity to Imatinib than other OCT (Fig. 1C). When investigating the roles of these transporters for the Imatinib accumulation in human RASF, we indeed identified hMATE1 to predominantly mediate this uptake whereas other OCTs had either no or only a minor (hOCTN1) influence on this process (Fig. 3A/B). The peak plasma levels observed in patients (between 4 and 10 µM depending on the daily dose [14], [15]) are clearly higher than the observed apparent affinity of hMATE1 for Imatinib. Furthermore, the uptake activity of hMATE1 directly governs anti-proliferative effects of Imatinib on hRASF as a blockade of hMATE1-mediated uptake abolished the inhibition of Imatinib on PDGF induced proliferation. This indicates the importance of TKI transport for therapeutic effects on RA (Fig. 3C).

After revealing the physiological transport, we further concentrated on pathology induced regulations and changes. RA is characterized by inflammatory processes that are known to impact on various cellular activities [12]. Therefore, the influence of the pro-inflammatory cytokines TNFα, IL-1β and IL-6, which have a great impact on RA, was analyzed. TNFα, IL-1β and IL-6 (+sIL-6R) decreased Imatinib uptake in hRASF by reducing hMATE1-mRNA and hMATE1 surface expression (Fig. 4). It has been shown that cytokines influence in various ways on the turnover of proteins [16]–[18], possibly explaining the shorter time that was needed to down-regulate hMATE1 protein with cytokines compared to siRNA. In consequence, inhibition of hMATE1 with pyrimethamine had no further influence on the Imatinib uptake as it had without prior cytokine stimulation. As expected by these findings, hMATE1 expression was reduced in hRASF compared to hOASF; however, possibly due to prior treatments of the patients or a partial loss of phenotype in cell culture, the difference did not reach significance (Fig. 2B). Another concomitant of inflammation is an acidic milieu as it is present in the synovial fluid of RA inflicted joints [13]. Imatinib transport in hRASF was decreased by an extracellular acidic milieu (Fig. 4A) corresponding to the functional properties of hMATE1 as also shown by the decreased Imatinib affinity of hMATE1 at acidic pH (Fig. S3). These findings indicate that the RA typical acidification combined with a reduction of hMATE1 expression by pro-inflammatory cytokines represents a critical factor for Imatinib mediated anti-proliferating effects, by decreasing its effective concentration in hRASF and consequently weakening its therapeutic effects. Furthermore, the regulation may explain why TKIs seem to be more effective in fibrotic diseases without immense inflammation like nephrogenic systemic fibrosis [19] compared to fibrotic diseases with inflammatory components like RA and SSc. Even though these results may suggest similar effects for other TKIs, it is important to note that different TKIs are not necessarily accumulated by the same transporters and mechanisms or affinities. Still, a similar effect for related drugs like p38 Map kinase inhibitors is certainly likely. Like TKIs, p38 MAP kinase inhibitors failed in clinical studies despite good *in vitro* effects [20]. In consequence a critical exploration of transport mediated drug processing is essential for developing new anti-inflammatory strategies and a reevaluation of known drugs may be indicated in certain cases.

## Materials and Methods

### Culture of Cell Lines

HEK293 cells (CRL-1573; American Type Culture Collection, Rockville, MD) were stably transfected with hMATE1-plasmid, a gift of Toshiya Katsura, and selected with 0.5 mg/ml hygromycin B (Invitrogen, San Diego, USA). hOCT1 stably transfected HEK293 cells were a gift of Prof. Koepsell, University of Würzburg. cDNAs of the novel organic cation transporter 1 (hOCTN1) and hOCTN2 subcloned into a doxycycline-inducible pEBTetD plasmid vector (a gift of Prof. Gründemann, University of Cologne) [21], [22] were transfected in HEK293 cells and transfected cells were selected with 3 mg/l puromycin (Invitrogen). Twenty-four hours before starting experiments OCTN expression was induced by 1 mg/l doxycycline (Sigma-Aldrich, Steinheim, Germany). Cells were grown at standard conditions. Transfection efficiency was controlled by quantitative real-time PCR (qRT-PCR).

### Culture of Synovial Fibroblasts (SF)

SF were isolated from rheumatoid arthritis (n = 10) and osteoarthritis (n = 4) tissues obtained during joint replacement surgery. Written informed consent of the patients was obtained. This procedure was specifically approved by the Ethics Committee of the University of Münster. RA patients met the American College of Rheumatology criteria. Isolated fibroblasts were cultured under standard conditions for a maximum of eight passages. When indicated, RA synovial fibroblasts (hRASF) were incubated with following cytokines at 10 ng/ml: TNFα, IL-1β, IL-6 and soluble IL-6 receptor (sIL-6R) (PeproTech, Hamburg, Germany). Cells were counted using CASYTT cell counter (Roche, Mannheim, Germany). Protein concentrations were assessed using Picodrop TM (Picodrop limited, Cambridge, UK).

### Determination of Apparent Affinities with 4-(4-(Dimethylamino)Styryl)-N-methylpyridinium (ASP^+^)

Apparent affinities of hOCT1, hOCTN1 and hMATE1 for Imatinib were determined in transfected HEK293 cells by inhibiting the ASP^+^ uptake (1 µM and 10 µM for hMATE1 only) with several Imatinib concentrations (10^−10^ to 10^−3^ M) as described elsewhere [23] at pH 7.4 and, only for hMATE1, at pH 6.4. ASP^+^ is substrate of these transporters (see [24], Fig. S1 and [25], respectively).

### HPLC Detection of Cellular Imatinib Accumulation

Cells were incubated for 10 min with 10 µM Imatinib in phosphate buffered saline (PBS) at 37°C or 4°C in the presence or not of specific inhibitors: 20 µM 1-methyl-4-phenylpyridinium iodide (MPP^+^), 40 µM (+)-ergothioneine and 200 nM pyrimethamine as specific inhibitors of hOCT1 (K_m = _15 µM, [26]), hOCTN1 (K_m = _21 µM, [27]) and hMATE1 (K_i = _77 nM, [28]), respectively. After incubation, cells were washed with ice-cold PBS and hypoosmotic lysis was induced with 0.1% formic acid. For Imatinib quantification the high pressure liquid chromatography (HPLC) method established by Widmer et al. [29] was used with modifications. The mobile phase consisted of (A) 0.1% formic acid and (B) acetonitrile and was delivered at 0.3 ml/min in a gradient program. After delivering 100% A for 7.4 min, a linear gradient to 50% B was applied within 12.4 min and maintained for 6 min. Thereafter isocratic elution with 100% B was applied for 6 min and then the column was re-equilibrated with 100% A for at least 6 min. The chromatographic system consisted of a P-900 pump and a UV-900 UV detector (Amersham Biosciences, Uppsala, Sweden) set to 261 nm for detection. Separation was performed on a Purospher STAR RP-18 column (55 mm×2.0 mm, 3 µm) (Merck, Darmstadt, Germany) equipped with a C18 guard column (4 mm×2.0 mm) (Phenomenex, Aschaffenburg, Germany) at room temperature. Instruments were piloted and data analyzed by the Unicorn 5.1 software (GE Healthcare, München, Germany).

### PCR

RNA was isolated with the Qiagen RNeasy Midikit (Qiagen, Gilden, Germany) and Invitrogen Super Script III system was used for reverse transcription. qRT-PCR was performed using SYBR Green PCR Master Mix and the ABI PRISM 7900 Sequence Detection System. Instruments and reagents were obtained from Applied Biosystems (Darmstadt, Germany). Gene expression is given in relation to the housekeeping-gene GAPDH. Standard PCR for hMATE1 was performed using a GreenGoTaq kit (Promega) with specific primer pairs as listed in Table S1. The reaction was started at 95°C for 2 min, followed by thermal cycling: 30 cycles of 30 s at 95°C, 30 s at 60°C and 60 s at 72°C. After the last cycle, an additional step of 10 min at 72°C was run. The PCR products were separated using agarose-gel electrophoresis and visualized using ethidium bromide staining.

### Immunofluorescence Staining

hRASF from 6 patients with or without prior cytokine cocktail incubation were fixed with 4% paraformaldehyd and prepared with 0.2% TritonX100 (Merck, Darmstadt, Germany). Unspecific binding was blocked with 10% bovine serum albumin before staining with a polyclonal anti-hMATE1 antibody (Sigma-Aldrich, diluted 1∶100) at 4°C overnight. Cells were incubated with a secondary Alexa Fluor 488 labeled donkey anti-goat-Ig antibody (Invitrogen, Karlsruhe, Germany, 1∶1000) for 45 min at room temperature and finally counterstained with DAPI. Antibody specificity was confirmed on hMATE1 transfected and WT-HEK293 cells (data not shown).

### Cell Proliferation Assay

Cell proliferation was evaluated by counting the cell number after incubation of hRASF with 10 ng/mL PDGF-AB for 24 hours. If indicated, cells were treated for 10 min. prior to PDGF-AB incubation with 2.5 µM Imatinib in the presence or not of 200 nM pyrimethamine to inhibit hMATE1 transport. Untreated hRASF served as control.

### hMATE1 Knockdown by siRNA

The hMATE1 specific siRNA (CGCUAAAUUGUCCAGGAAAdTdT) was obtained from Sigma (Sigma-Aldrich, Steinheim, Germany). The nonsilencing control siRNA (Stealth RNAi Neg. Control, medium GC content) was obtained from Invitrogen. RASF were transfected with 50 nM siRNA using N-TER Nanoparticle siRNA transfection system (Sigma-Aldrich) according to manufacturer’s instructions. Due to the stable expression of hMATE1 protein, transfection was repeated every 48 h and subsequent experiments were done 192 h after the first siRNA transfection. Knockdown efficiencies were quantified at protein level by Western blot.

### Surface Biotinylation

For quantitative analysis of hMATE1 surface expression, surface proteins of hRASF monolayers were isolated using a commercial kit (Pierce Cell Surface Protein Isolation Kit, Thermo Scientific, Rockford, IL, USA). Briefly, cell monolayers were biotin labeled, quenched, sonicated, lysed, and clarified by centrifugation according to the manufacturer’s protocol. To isolate biotin-labeled protein, lysates were then incubated with immobilized NeutrAvidin TM gel, paying special attention to load the same protein quantity on the gel. The gel was washed and then incubated 1 h with SDS-PAGE sample buffer including 50 mM dithiothreitol (DTT). Eluates were analyzed for hMATE1 by immunoblotting using polyvinylidene fluoride (PVDF) membranes and a polyclonal anti-hMATE1 antibody (abcam, Cambridge, UK) diluted 1∶100. Immunoreactive bands were detected with horseradish peroxidase (HRP)-conjugated secondary antibodies (Dako, Hamburg, Germany) and enhanced chemiluminescence.

### Chemicals

Imatinib was purchased from LC Laboratories (Woburn, USA). Pyrimethamine, L-(+)-ergothioneine, MPP^+^, and PDGF-AB were obtained from Sigma. ASP^+^ was purchased from Molecular Probes (Invitrogen). Chemicals were dissolved in PBS or HCO_3_-free Ringer-like solution. Dimethylsulphoxide was added for pyrimethamine but the final concentration did not affect experimental results (data not shown).

### Statistical Analysis

Data were analyzed using GraphPad Prism, Version 4.0 (GraphPad Software, Inc., San Diego, USA) and are shown as mean ± SEM. For experiments using HEK293 cells the number of observations is given in brackets. In HPLC and qRT-PCR experiments with human samples the numbers in brackets refer to the number of observations/tested patients or tested patients, respectively. When indicated, ANOVA with Tukey posthoc test or a paired Student t test was applied. A P-value<0.05 was considered statistically significant.

## Supporting Information

Figure S1 **ASP^+^ is a substrate for hOCTN1.** Comparison of ASP^+^ uptake by HEK293 cells stably transfected with doxycycline-inducible pEBTetD/hOCTN1 plasmid vector cultured with (hOCTN1 “on”) or without (hOCTN1 “off”) 1 µg/ml doxycycline for 24 h. Results are expressed as % of the ASP^+^ uptake observed in hOCTN1 “off” cells. Values are mean ± SEM. * indicates statistically significant effects (P<0.05). The number of experiments is indicated above the column.
(PDF)

Figure S2 **Incubation of hRASF with pro-inflammatory cytokines for less than 18 hours does not influence Imatinib transport.** Time dependent influence of a TNFα, IL-1β and IL-6 (+sIL-6R) cocktail (each at 10 ng/ml) on specific Imatinib uptake (10 µM) in hRASF given as difference of accumulation at 4°C and 37°C. Results show number of observations/patients in brackets. Values are mean ± SEM.
(PDF)

Figure S3 **An acidic pH reduces the apparent affinity of hMATE1 to Imatinib.** Apparent affinity of Imatinib on hMATE1 stable expressed in HEK293 cells at extracellular pH 6.4 measured by concentration dependent inhibition of ASP^+^ uptake (IC_50_ = 4 µM). Values are mean ± SEM.
(TIF)

Table S1 **Primer Sequences used in this study.**
(PDF)
