# Supplementary figures and images for: Transport Mechanisms and Their Pathology-Induced Regulation Govern Tyrosine Kinase Inhibitor Delivery in Rheumatoid Arthritis
Source: PLoS One. 2012 Dec 20;7(12):e52247. doi: 10.1371/journal.pone.0052247 (PMC3527388; doi:10.1371/journal.pone.0052247)

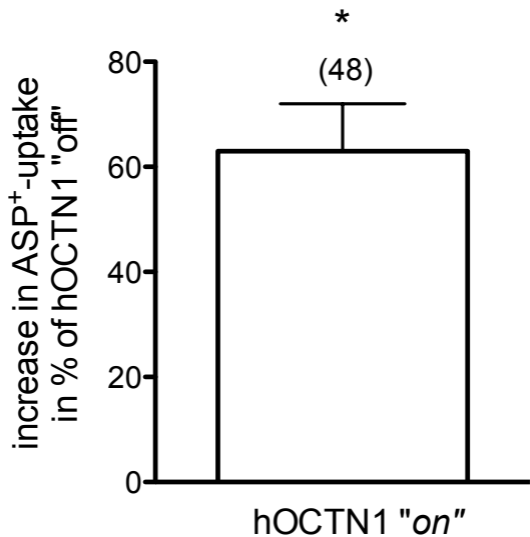

Supplement: Figure S1 — ASP+ is a substrate for hOCTN1. Comparison of ASP+ uptake by HEK293 cells stably transfected with doxycycline-inducible pEBTetD/hOCTN1 plasmid vector cultured with (hOCTN1 “on”) or without (hOCTN1 “off”) 1 µg/ml doxycycline for 24 h. Results are expressed as % of the ASP+ uptake observed in hOCTN1 “off” cells. Values are mean ± SEM. * indicates statistically significant effects (P<0.05). The number of experiments is indicated above the column. (PDF) [file pone.0052247.s001.pdf]

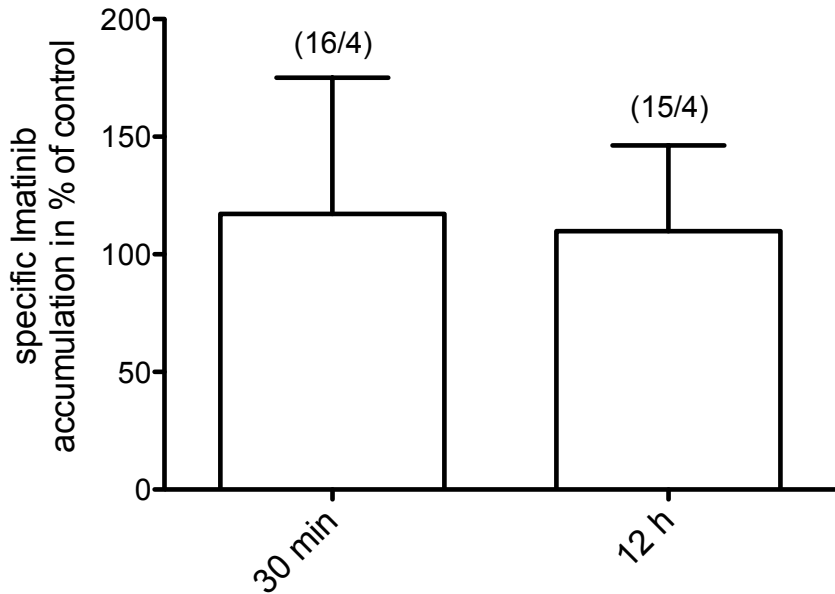

Supplement: Figure S2 — Incubation of hRASF with pro-inflammatory cytokines for less than 18 hours does not influence Imatinib transport. Time dependent influence of a TNFα, IL-1β and IL-6 (+sIL-6R) cocktail (each at 10 ng/ml) on specific Imatinib uptake (10 µM) in hRASF given as difference of accumulation at 4°C and 37°C. Results show number of observations/patients in brackets. Values are mean ± SEM. (PDF) [file pone.0052247.s002.pdf]

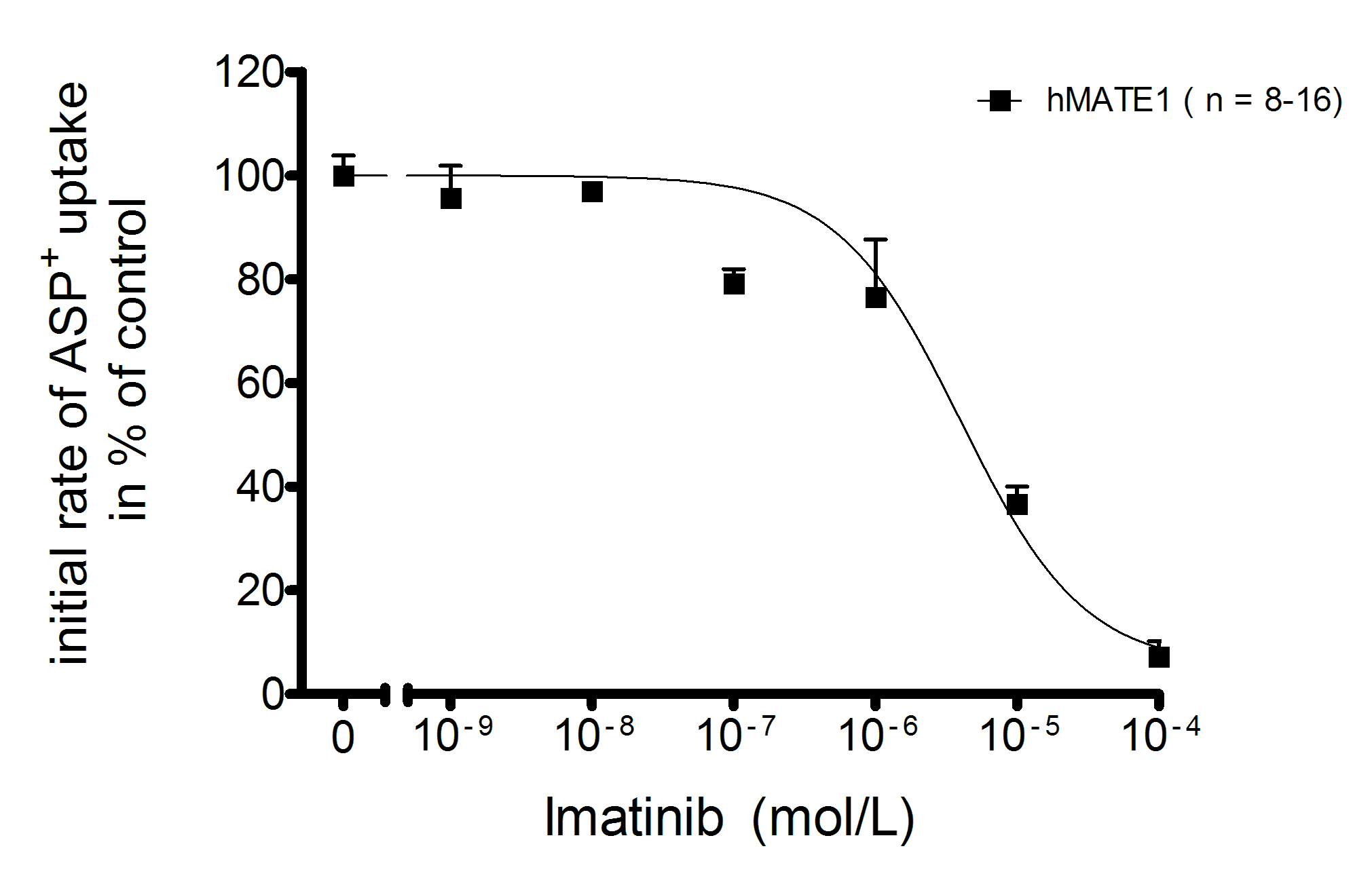

Supplement: Figure S3 — An acidic pH reduces the apparent affinity of hMATE1 to Imatinib. Apparent affinity of Imatinib on hMATE1 stable expressed in HEK293 cells at extracellular pH 6.4 measured by concentration dependent inhibition of ASP+ uptake (IC50 = 4 µM). Values are mean ± SEM. (TIF) [file pone.0052247.s003.tif]
