# Supplementary material for: Transport Mechanisms and Their Pathology-Induced Regulation Govern Tyrosine Kinase Inhibitor Delivery in Rheumatoid Arthritis
Source: PLoS One. 2012 Dec 20;7(12):e52247. doi: 10.1371/journal.pone.0052247 (PMC3527388; doi:10.1371/journal.pone.0052247)
Supplement: Table S1 — Primer Sequences used in this study. (PDF) [file pone.0052247.s004.pdf]

Gene name	Primer sequence (5' → 3')	Accession no.
glyceraldehydes-3-phosphate-dehydrogenase (GAPDH)	*S* CAA GCT CAT TTC CTG GTA TGA C<br>*AS* GTG TGG TGG GGG ACT GAG TGT GG	BC013852
solute carrier family 22, member 1 (SLC22A1, hOCT1)	*S* CAT CAT AAT CAT GTG TGT TGG CC<br>*AS* CAA ACA AAA TGA GGG GCA AGG CTT	NM_003057
solute carrier family 22, member 2 (SLC22A2, hOCT2)	*S* CAT TGA ACT AAG AAG AGA GAC CG<br>*AS* CCA CAG TGT ACA ATA GAC TCC A	NM 153191
solute carrier family 22, member 4 (SLC22A4, hOCTN1)	*S* TC CAG AAA CCT TAG AGC AGA TGC<br>*AS* GA ATG CAG TTA TTA GAA CCT TGG G	NM_003059
solute carrier family 22, member 5 (SLC22A5, hOCTN2)	*S* GTA CCC CAC TCC CAG ACA CC<br>*AS* GCT GTG CTT TTA AGG ATT GTG GG	NM_003060
solute carrier family 47, member 1 (SLC47A1, hMATE1)	*S* GCA ACC ACA CTT GGA GTG ATG G<br>*AS* GAG CAG AAT TCC CAC TCC GAG	NM_018242
S: Sense; AS: Antisense		
